# Myosteatosis in a systemic inflammation‐dependent manner predicts favorable survival outcomes in locally advanced esophageal cancer

**DOI:** 10.1002/cam4.2593

**Published:** 2019-10-01

**Authors:** Camila T. B. Gabiatti, Mariane C. L. Martins, Daniela L. Miyazaki, Leandro P. Silva, Fabiana Lascala, Ligia T. Macedo, Maria Carolina Santos Mendes, José Barreto Campello Carvalheira

**Affiliations:** ^1^ Division of Oncology Department of Internal Medicine Faculty of Medical Sciences State University of Campinas (UNICAMP) Campinas SP Brazil

**Keywords:** cachexia, esophageal neoplasms, myosteatosis, sarcopenia, survival analysis

## Abstract

Increased adiposity and its attendant metabolic features as well as systemic inflammation have been associated with prognosis in locally advanced esophageal cancer (LAEC). However, whether myosteatosis and its combination with systemic inflammatory markers are associated with prognosis of esophageal cancer is unknown. Our study aimed to investigate the influence of myosteatosis and its association with systemic inflammation on progression‐free survival (PFS) and overall survival (OS) in LAEC patients treated with definitive chemoradiotherapy (dCRT). We retrospectively gathered information on 123 patients with LAEC submitted to dCRT at the University of Campinas Hospital. Computed tomography (CT) images at the level of L3 were analyzed to assess muscularity and adiposity. Systemic inflammation was mainly measured by calculating the neutrophil‐to‐lymphocyte ratio (NLR). Median PFS for patients with myosteatosis (n = 72) was 11.0 months vs 4.0 months for patients without myosteatosis (n = 51) (hazard ratio [HR]: 0.53; 95% confidence interval [CI], 0.34‐0.83; *P* = .005). Myosteatosis was also independently associated with a favorable OS. Systemic inflammation (NLR > 2.8) was associated with a worse prognosis. The combination of myosteatosis with systemic inflammation revealed that the subgroup of patients with myosteatosis and without inflammation presented less than half the risk of disease progression (HR: 0.47; 95% CI: 0.26‐0.85; *P* = .013) and death (HR: 0.39; 95% CI, 0.21‐0.72; *P* = .003) compared with patients with inflammation. This study demonstrated that myosteatosis without systemic inflammation was independently associated with favorable PFS and OS in LAEC patients treated with dCRT.

## Graphical abstract

For the first time, this study evaluated the impact of myosteatosis and its association with systemic inflammation on outcomes among 123 patients treated with definitive chemoradiotherapy for locally advanced esophageal cancer. In this kind of cancer population with mechanical obstruction of digestive tract, myosteatosis without systemic inflammation predicted favorable prognosis. These results underscore potentially modifiable biomarkers of prognosis and therefore may contribute to improve nutritional and medical strategies in this setting of patients.

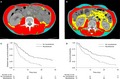

## 1 INTRODUCTION

Despite the recent advances in esophageal cancer treatment, this malignancy is still associated with a poor prognosis even before it evolves to advanced stage.^1^ Clinically, this neoplasm is characterized by digestive tract obstruction, which results in malnutrition and its accompanying body composition changes.^2^ Given that weight loss is reported in approximately 79% of esophageal cancer patients and maintenance of nutrition is one of the biggest treatment challenges,^2^ it is not surprising that markers of nutritional status are associated with prognosis. For instance, preoperative severe weight loss has been associated with decreased overall survival (OS) after esophageal cancer resection.^3^ Interestingly and in opposition to other malignancies, diabetes was associated with better prognosis.^4^ Moreover, a large prospective study showed a strong relationship between high body mass index (BMI) and fewer deaths from squamous cell carcinoma of the esophagus.^5^ Thus, the identification of nutritional‐related features that predict esophageal cancer clinical outcomes has the potential to improve treatment strategies by allowing a patient‐personalized therapeutic plan.

Cachexia syndrome, and its attendant complications, is one of the most prevalent causes of death in cancer patients.^6^ Computed tomography (CT) evaluation of adiposity and muscularity measured at the level of the third lumbar vertebra is highly correlated with body composition^7^ and has become a standard tool for caquexia assessment.^8^ Sarcopenia and decreased muscle radiodensity are the most prominent studied parameters in CT evaluations. Sarcopenia is a key feature of cachexia syndrome,^9^ as such it is a predictor of poorer OS as well as increased cancer recurrence in distinct cancers.^10^, ^11^, ^12^ Accordingly, sarcopenia is correlated with higher rates of treatment‐related complications^13^, ^14^ and poor prognosis in surgically treated esophageal cancer.^15^ In contrast, it was not yet associated with survival outcomes in metastatic settings.^16^ Decreased muscle radiodensity, mainly caused by intramyocellular triglycerides (myosteatosis), is also associated with poor survival outcomes in distinct tumours.^12^, ^17^, ^18^, ^19^ Importantly, myosteatosis is not directly biologically correlated with sarcopenia, nor is part of cachexia definition.^20^ Nonetheless, whether myosteatosis predicts esophageal cancer survival outcomes has not been assessed yet.

Cancer cachexia is mediated by complex host‐tumor cross‐talks that results in a series of tumor‐secreted proinflammatory factors, leading to anorexia as well as muscle weaning and lower adipose tissue depots.^6^ Consistently, a recent report evaluating the combined analysis of body composition and systemic inflammatory indexes demonstrated that colorectal cancer patients with sarcopenia and inflammation (neutrophil‐to‐lymphocyte ratio [NLR] higher than 3) had twofold increase in the risk of death.^10^

In contrast to the canonical pathophysiology of cancer cachexia, locally advanced esophageal cancer (LAEC)‐mediated mechanical obstruction of digestive tract triggers a unique kind of cancer cachexia, since the mechanical obstruction is the preponderant pathophysiological factor for its development.^2^ Congruent with a low ingestion, malnutrition was more frequently reported in individuals with esophageal cancer in a study that evaluated 1000 patients with different cancer types.^21^ However, a systematic characterization of body composition and its combination with systemic inflammatory indexes influence on survival outcomes of LAEC treated with definitive chemoradiotherapy (dCRT) is yet to be determined. Therefore, in the present analysis, we retrospectively evaluated myosteatosis and sarcopenia as well as systemic inflammatory indexes as possible prognostic factors in patients with esophageal carcinoma.

## 2 MATERIAL AND METHODS

### 2.1 Patients and procedures

In this retrospective study, between January 2010 and December 2016, we identified a total of 181 patients diagnosed with LAEC treated at Campinas State University Hospital. Inclusion criteria were as follows: (a) histologically confirmed esophageal squamous cell carcinoma or adenocarcinoma; (b) patients submitted to dCRT; (c) abdominal CT scans performed within 4 months of diagnosis that were assessed electronically in the Picture Archiving and Communication System; and (d) availability of clinical, demographic, and anthropometric data of interest. Patients who underwent esophagectomy, received chemotherapy or radiotherapy exclusively, with a second cancer not in esophagus, or with death event occurring up to 30 days following diagnosis, were excluded from this study.

Medical records were considered for data collection from the diagnosis date until the last date of follow‐up or death. Clinical evaluation and assessment of abdominal and thoracic CTs were performed routinely for follow‐up for progression‐free survival (PFS). Eastern Cooperative Oncology Group (ECOG) performance status and BMI were determined by functional status and anthropometric measurements (height and weight), respectively, checked by hospital staff.

This study was approved by the Campinas State University Institutional Review Board (2.239.135), with a waiver of informed consent.

### 2.2 Body composition

The CT scans used for analysis were carried out as part of diagnostic and staging purposes. Muscle area, muscle radiodensity, and adiposity were measured from CT scans within 4 months of diagnosis and before chemotherapy and radiation (median [range], 1.1 [−3.2 to 3.8] months after diagnosis), by a single trained researcher who was blinded to outcome assessment, using Slice‐OMatic Software, version 5.0 (Tomovision™).^8^, ^22^, ^23^

The average of two consecutive axial images at the level of L3 was considered for body composition quantification, including total skeletal muscle (SM), visceral (VAT) and intramuscular adipose tissue (IMAT) cross‐sectional areas, and mean muscle attenuation (MA). Tissue areas were identified by their anatomic features and quantified according to the standard Hounsfield unit (HU) range of −29 to 150 for SM, −150 to −50 for VAT, and −190 to −30 for IMAT and subcutaneous adipose tissue.^24^, ^25^

Skeletal muscle index (SMI), visceral fat index (VFI), and subcutaneous fat index (SFI) were calculated from total adipose and muscle mass cross‐sectional area divided by height square (cm^2^/m^2^). The MA in HU was also reported for the whole muscle area at L3. As previously described by Martin et al, the following parameters were used to define sarcopenia (SMI < 41 cm^2^/m^2^ for women; SMI < 43 cm^2^/m^2^ if BMI < 25 kg/m^2^; and SMI < 53 cm^2^/m^2^ if BMI ≥ 25 kg/m^2^ for men) and myosteatosis (MA < 41 HU if BMI < 25 kg/m^2^ and MA < 33 HU if BMI ≥ 25 kg/m^2^).^22^ Visceral obesity was established as VAT > 80.1 cm^2^ for females and VAT > 163.8 cm^2^ for males.^26^

### 2.3 Systemic inflammatory indexes

Complete blood count routinely collected for initiation of chemotherapy was used to calculate NLR by dividing neutrophils by lymphocyte absolute counts. We categorized this index using the median value, which fell in a meaningful clinical value of 2.8.^27^ Likewise, platelet‐to‐lymphocyte ratio (PLR) was obtained using platelets in the numerator of the previous ratio instead of neutrophils. For analysis, this variable was also categorized using the median value (133), which fell in a range that is often used in other studies to categorize this inflammatory index.^28^

### 2.4 Treatment toxicity

All data on treatment toxicity (eg, hematologic disorders, nausea, vomit, diarrhea, etc) were reviewed from medical records. Treatment toxicity was dichotomized into present or absent and any type of grade 3 or 4 toxicity according to the Common Terminology Criteria for Adverse Events [CTCAE v4.03]).^29^ Carboplatin plus paclitaxel or 5‐fluorouracil associated with cisplatin was selected at the discretion of the attendant physician as the first‐line chemotherapy regimen and radiotherapy at a median dosage of 50 Gy.

### 2.5 Endpoints

The co‐primary endpoints were OS (calculated by time between LAEC diagnosis and death from any cause) and PFS (two distinct calculations: time between the diagnosis and disease progression or death; or the date of performed CT and disease progression or death) and response rate. In the event of patients who were still alive, censoring occurred at the last follow‐up date registered in the medical record.

### 2.6 Statistical analysis

The relationships between myosteatosis and continuous variables were assessed by Student's t test (presented as mean ± SD) or Wilcoxon rank‐sum test (presented as median ± interquartile range^30^) for parametric and nonparametric distributions, respectively. Categorical variables were presented as proportions and analyzed by Chi‐square or Fisher exact tests, depending on the distribution of the variable. Kaplan‐Meier curves, log‐rank tests, univariate and multivariate Cox proportional hazards were applied to analyze the impact of myosteatosis and sarcopenia on survival outcomes. To identify variables that interfered in outcomes, all values *P* < .1 in the univariate analyses were included in the multivariate Cox regression model. Sensitivity analysis was performed to reduce the probability of reverse causality excluding patients who died within 3 months after the diagnosis of LAEC. Effect modification analyses were then performed, aiming for subgroup differences. Overall survival and PFS were evaluated using the nonparametric Kaplan‐Meier method. All statistical analyses were performed using Stata software, version 12.0 (StataCorp LP^®^). Statistical significance was established with two‐sided *P* value < .05.

## 3 RESULTS

### 3.1 Patient and body composition characteristics

Among the 181 patients treated for LAEC between January 2010 and December 2016, 123 patients met the inclusion criteria. Median follow‐up time was 10.1 months (IQR: 3.7‐23.6 months).

**Figure 1:**
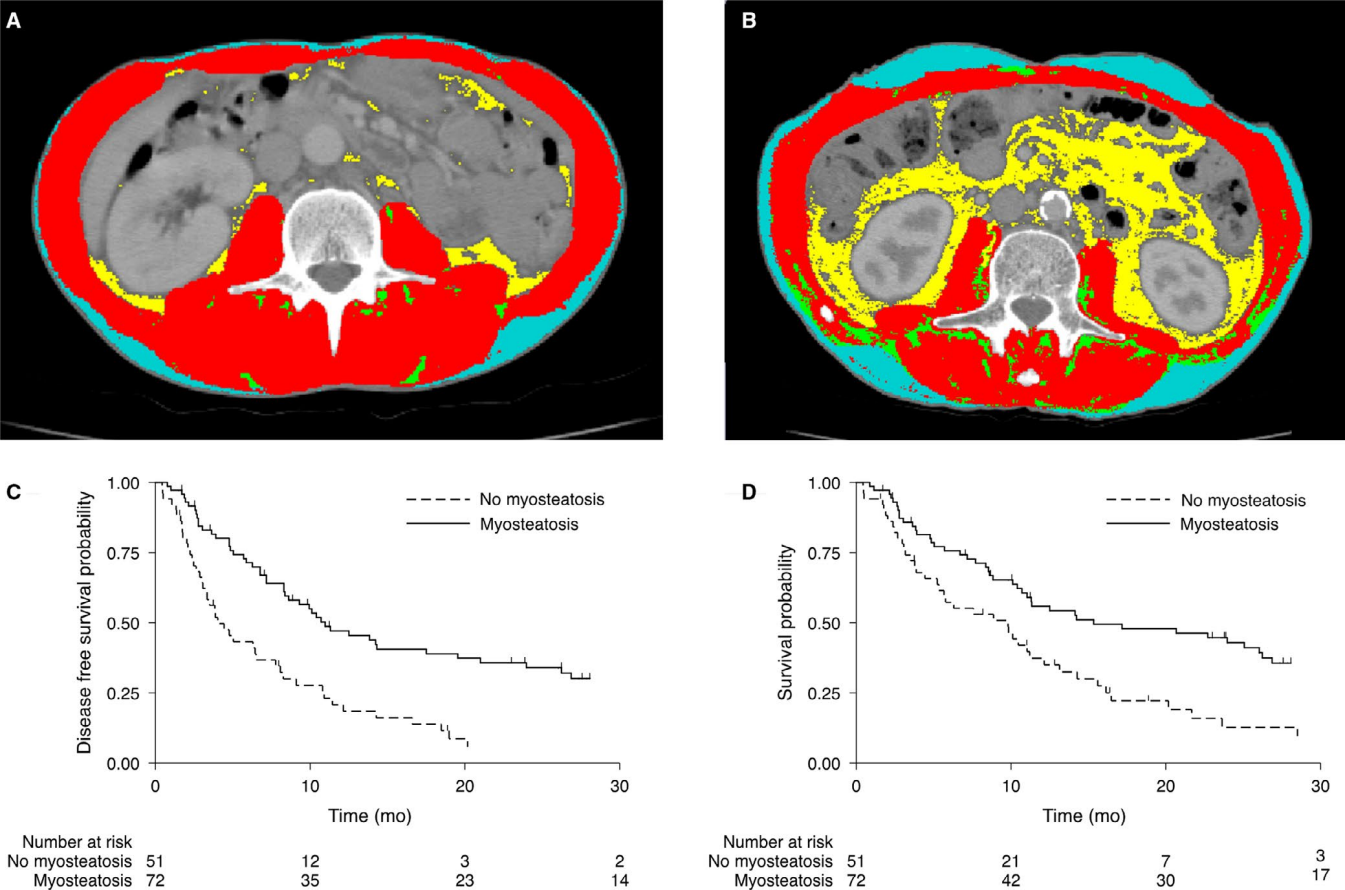
Representative computed tomography images in patients with (A—43 y old man with squamous cell carcinoma; BMI = 19.9; NLR = 8.8; PLR = 1871.0) and without (B—57 y old man with squamous cell carcinoma; BMI = 21.3; NLR = 2.4; PLR = 67.3) myosteatosis with LAEC treated with dCRT. PFS (C) and OS (D) in patients with and without myosteatosis with LAEC treated with dCRT. Color legend: Subcutaneous (blue), visceral (yellow), intramuscular adipose tissue (green), and skeletal muscle mass (red). BMI, body mass index; dCRT, definitive chemoradiotherapy; LAEC, locally advanced esophageal cancer; NLR, neutrophil‐lymphocyte ratio; PLR, platelet‐to‐lymphocyte ratio

We detected myosteatosis in 72 patients (58.5%). Patients with myosteatosis were older, had a higher BMI, and lost less weight compared to non‐myosteatosis individuals (Table 1). Interestingly, as shown in Table 2, patients with myosteatosis presented higher visceral, subcutaneous, and intramuscular adipose tissue depots compared to subjects without myosteatosis (Figure 1). We did not observe a difference in sarcopenia distribution between patients with or without myosteatosis.

**Table 1:** Selected characteristics according to myosteatosis of esophageal cancer patients

Characteristic	All‐patients (n = 123)	Non‐myosteatosis (n = 51)	Myosteatosis (n = 72)	*P* value
Age, mean (SD), y	59.3 (11.7)	56.1 (9.9)	61.6 (12.3)	.01
Sex, no (%)				
Male	107 (87.7)	45 (88.2)	63 (87.5)	.90
Female	15 (12.3)	6 (11.8)	9 (12.7)	
Body mass index (kg/m^2^), no (%)				
<18.5	41 (33.3)	25 (49.0)	16 (22.2)	.02
18.5‐24.9	66 (53.7)	21 (41.2)	45 (62.5)	
25‐30	13 (10.6)	4 (7.8)	9 (12.5)	
>30	3 (2.4)	1 (2.0)	2 (2.80)	
Weight loss, no (%)				
<5	8 (6.5)	3 (5.9)	5 (6.9)	.04
5‐9.9	22 (17.9)	4 (7.8)	18 (25.0)	
>10	93 (75.6)	44 (88.3)	49 (68.1)	
Hypertension, no (%)	36 (29.3)	11 (21.6)	25 (34.7)	.11
Dyslipidemia, no (%)	4 (3.2)	2 (3.9)	2 (2.7)	.55
Diabetes, no (%)	5 (4.1)	2 (3.9)	3 (4.2)	.66
Histology, no (%)				.32
Adenocarcinoma	11 (8.9)	3 (5.9)	8 (11.1)	
Squamous cell carcinoma	112 (91.1)	48 (94.1)	64 (88.9)	
Tumor location, no (%)				
Upper third	15 (12.2)	3 (5.9)	12 (16.6)	.19
Middle third	71 (57.7)	32 (62.7)	39 (54.2)	
Lower third	37 (38.1)	16 (31.4)	21 (29.2)	
Chemotherapy, no (%)				
5‐Fluorouracil + cisplatin	19 (15.5)	7 (13.7)	12 (16.7)	.88
Carboplatin + paclitaxel	102 (82.9)	43 (84.3)	59 (81.9)	
Others	2 (1.6)	1 (2.0)	1 (1.4)	
Toxicity grade III‐IV, no (%)				
No	31 (25.2)	9 (17.7)	22 (30.6)	.10
Yes	92 (74.8)	42 (82.3)	50 (69.4)	
ECOG, no (%)				
0	58 (47.5)	22 (43.1)	36 (47.5)	.33
1	60 (49.2)	26 (51.0)	60 (49.2)	
2	4 (3.3)	3 (5.9)	4 (3.3)	

Abbreviations: ECOG, Eastern Cooperative Oncology Group Performance; SD, standard deviation.

**Table 2:** Body composition and inflammatory indexes according to myosteatosis of esophageal cancer patients

Parameter	All‐patients (n = 123)	No‐myosteatosis (n = 51)	Myosteatosis (n = 72)	*P* value
Skeletal muscle, mean (SD)				
Area (cm^2^)	122.1 (24.5)	118.0 (27.2)	125.0 (22.0)	.12
Mean MA (HU)	38.8 (9.3)	47.1 (6.4)	32.9 (5.7)	<.01
SMI (cm^2^/m^2^)	44.7 (8.4)	43.2 (9.3)	45.7 (7.7)	.10
Sarcopenia, no (%)	57 (46.3)	24 (47.1)	33 (45.8)	.89
Adipose tissue, median (IQR)				
Visceral, area (cm^2^)	25.1 (6.7‐102.0)	8.4 (1.3‐21.5)	65.9 (21.5‐135.2)	<.01
VFI (cm^2^/m^2^)	8.6 (2.3‐38.3)	2.9 (0.6‐7.9)	25.4 (7.7‐52.3)	<.01
Subcutaneous, area (cm^2^)	40.9 (13.6‐75.3)	19.0 (0.9‐48.3)	52.7 (29.0‐104.0)	<.01
SFI (cm^2^/m^2^)	15.1 (4.6‐28.7)	6.9 (0.4‐17.9)	19.1 (10.5‐38.1)	<.01
Intramuscular, area (cm^2^)	6.6 (2.9‐11.2)	2.9 (1.9‐6.5)	9.3 (5.7‐12.7)	<.01
Inflammatory indexes				
NLR, median (IQR)	2.8 (2.3)	3.8 (3.0)	2.6 (1.9)	<.01
PLR, median (IQR)	133.1 (81.7)	153.7 (71.0)	118.4 (76.0)	<.01

Abbreviations: HU, Hounsfield units; IQR, interquartile range; MA, muscle attenuation; NLR, neutrophil‐to‐lymphocyte ratio; SD, standard deviation; SFI, subcutaneous fat index; SMI, skeletal muscle index; VFI, visceral fat index.

### 3.2 Survival analysis

**Figure 2:**
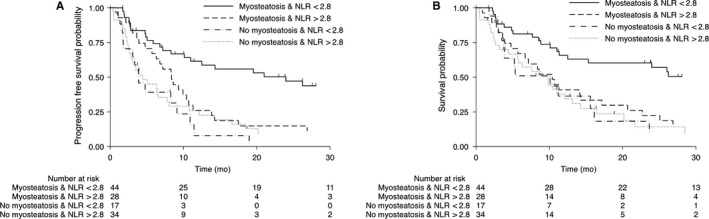
Progression‐free survival (A) and overall survival (B) according to neutrophil‐to‐lymphocyte ratio and myosteatosis in patients with locally advanced esophageal cancer treated with definitive chemoradiotherapy

Although we did not detect differences in response rate (partial + complete response) between patients with (57.8%) or without (67.2%) myosteatosis (*P* = .23), Kaplan‐Meier curves demonstrated that patients with myosteatosis presented better PFS (log‐rank *P* < .001) (Figure 2A). A significantly greater PFS related to myosteatosis was detected according to adjusted Cox regression analysis (HR: 0.53, 95% CI, 0.34‐0.83; *P* = .005). Median PFS was 4.0 months in the non‐myosteatosis group vs 11.0 months for myosteatosis individuals (Table 3). The above‐described results were similar when PFS was calculated using as reference the date of performed CT instead of diagnosis date (Table S1).

**Table 3:** Myosteatosis and survival from date of treatment start

Parameter	No‐myosteatosis (n = 51)	Myosteatosis (n = 72)	*P* value
Progression free survival			
#Events/at risk	44/51	53/72	
Median (mo)	4.0	11.0	
Age‐adjusted	Referent	0.49 (0.32‐0.75)	.001
Adjusted^a^	Referent	0.53 (0.34‐0.83)	.005
Overall survival			
#Events/at risk	41/51	49/72	
Median (mo)	9.8	15.3	
Age‐adjusted	Referent	0.58 (0.38‐0.89)	.013
Adjusted^a^	Referent	0.57 (0.36‐0.91)	.018

Abbreviations: BMI, body mass index; ECOG, Eastern Cooperative Oncology Group Performance.

a Cox model adjusted for age (continuous), weight loss (<5%, 5‐9.9, or >9), BMI (<18.5, 18.5‐24.9, 25‐30, or >30), and ECOG (0, 1, or 2).

Analysis of OS also showed statistically significant differences between the myosteatosis and non‐myosteatosis groups. Kaplan‐Meier curves demonstrated that patients with myosteatosis presented better OS compared to those without myosteatosis (log‐rank *P* = .005) (Figure 2B). As shown in Table 3, a significantly greater OS related to myosteatosis was detected according to adjusted Cox regression analysis (HR: 0.57; 95% CI, 0.36‐0.91; *P* = .018). Median OS was 9.8 months in the non‐myosteatosis group vs 15.3 months for individuals with myosteatosis (Table 3). Two‐year OS rates were 7.8% and 33.3% in the non‐myosteatosis and myosteatosis groups, respectively. The above‐described results were similar when OS was calculated using as reference the date of performed CT instead of diagnosis date (Table S1).

Given that body composition alterations could be associated with early mortality, we performed sensitivity analyses to evaluate the possibility of reversed causality. The PFS analysis excluding the patients in whom disease progressed within the first 3 months (31 patients) showed persistent statistical significance (HR: 0.58; 95% CI, 0.33‐0.99; *P* < .049). Similar results were obtained excluding the 23 patients who died within the first 3 months. Myosteatosis predicted increased OS (HR: 0.54; 95% CI, 0.31‐0.93; *P* = .026).

Kaplan‐Meier curves also revealed that individuals with sarcopenia had similar PFS and OS compared with non‐sarcopenia group (Figure S1). We failed to detect that SM area and SMI were associated with survival outcomes (Table S2). A higher visceral fat area and VFI were associated with better prognosis, in continuous analyses (Table S2). While, a NLR less than 2.8 was associated with decreased risk of disease progression (HR: 0.64; 95% CI, 0.42‐0.98; *P* = .042) and death (HR: 0.56; 95% CI, 0.36‐0.88; *P* = .012) (Table S2). Similarly, a PLR less than 133 was associated with improved prognosis (Table S2).

### 3.3 Subgroup analysis by inflammatory indexes

There are some reasons to hypothesize that lower inflammatory indexes could discriminate a subgroup of LAEC patients where myosteatosis is not induced by cancer‐mediated inflammation and consequently this subgroup would have an increased survival. Firstly, increased inflammatory indexes are associated not only with cancer but also with increased visceral adiposity.^31^ Secondly, the absolute value of these indexes is lower in metabolic diseases than that in cancer.^31^, ^32^ Finally, both visceral adiposity and cancer are associated with myosteatosis.^33^ In accordance with this hypothesis, subgroup analysis showed interaction between myosteatosis impact on survival outcomes (PFS and OS) and NLR < 2.8 (*P* < .001), with patients who have NLR < 2.8 showing a more favorable prognosis, which was confirmed by multivariable analysis (Table 4). Whereas, patients with myosteatosis and NLR > 2.8 presented a very poor prognosis similar to patients without myosteatosis (Figure 2; log‐rank *P* < .001).

**Table 4:** Myosteatosis, neutrophil‐to‐lymphocyte ratio, and survival from date of treatment start

	Progression‐free survival				Overall survival			
	No myosteatosis		Myosteatosis		No myosteatosis		Myosteatosis	
	NLR < 2.8	NLR > 2.8	NLR < 2.8	NLR > 2.8	NLR < 2.8	NLR > 2.8	NLR < 2.8	NLR > 2.8
#Events/at risk	29/34	15/17	26/44	27/28	27/34	14/17	26/44	23/28
Median (mo)	4.4	3.9	24.0	8.4	9.8	10.1	30.8	10.4
HR	1.328		0.469		1.178		0.388	
95% CI	0.698‐2.526		0.259‐0.851		0.611‐2.270		0.208‐0.724	
*P*	.388		.013		.625		.003	

Note

Cox model adjusted for age (continuous), weight loss (<5%, 5‐9.9, or >9), BMI (<18.5, 18.5‐24.9, 25‐30, or >30), and ECOG (0, 1, or 2).

Abbreviations: BMI, body mass index; ECOG, Eastern Cooperative Oncology Group Performance; NLR, neutrophil‐to‐lymphocyte ratio.

Among patients with myosteatosis, characteristics were similar between systemic inflammation‐categorized subgroups, except that patients with NLR < 2.8 tended to be ECOG 0, while patients with NLR > 2.8 tended to be ECOG 1 (*P* = .001; Table S3). Patients with NLR < 2.8 had an improved PFS (HR: 0.47; 95% CI: 0.26‐0.85; *P* = .013) and OS (HR: 0.39; 95% CI, 0.21‐0.72; *P* = .003) (Table 4). Consistently, the survival outcomes analysis using standard cutoffs for low (<3) and high (>5) NLR showed persistent statistical significance.^32^ Among patients with NLR < 3, myosteatosis predicted increased PFS (HR: 0.34; 95% CI, 0.17‐0.68; *P* = .002) and OS (HR: 0.43; 95% CI, 0.26‐0.88; *P* = .021). Whereas, among patients with NLR > 5, myosteatosis did not affect PFS (HR: 0.59; 95% CI, 0.17‐2.26; *P* = .048) and OS (HR: 0.48; 95% CI, 0.18‐2.83; *P* = .72).

We obtained similar results when systemic inflammation subgroups were categorized by PLR (Figure S2; Table S4).

## 4 DISCUSSION

In this retrospective study, we found that myosteatosis was significantly associated with favorable PFS and OS in patients submitted to dCRT for LAEC, suggesting that myosteatosis is a biomarker that may help to implement personalized nutritional and therapeutic approaches. Furthermore, increased NLR was more frequently observed in patients without myosteatosis and subgroup analysis revealed that the co‐occurrence of myosteatosis and low NLR predicted a less than half disease progression and mortality risks in LAEC patients treated with dCRT.

The European Society for Parenteral and Enteral nutrition guidelines strongly recommend that patients submitted to esophageal radiotherapy should be ensured an adequate nutritional support, which should include the use of nutritional supplements.^34^ Interestingly, we observed a low prevalence of overweight and obesity in our population (15.3%). Furthermore, we did not detect visceral obesity and 93% of our cohort presented more than 5% weight loss. It is important to note that our data are in contrast to the prevalence of overweight and obesity, which reaches rates of up to 68%, in studies where adenocarcinoma was the main histological type and the patients were submitted to esophagectomy.^35^ Altogether, these data suggest that individuals submitted to dCRT for esophageal cancer are extremely vulnerable and should have personalized nutritional counseling.

In striking contrast to previous reports,^12^, ^17^, ^18^, ^19^ our data show that myosteatosis predicted a favorable prognosis. The pathophysiological mechanisms associated with increased intramyocellular lipid deposits with cancer‐mediated weight loss are still not clear^36^; however, enhanced lipolysis, insulin resistance, and impaired mitochondrial oxidation are often implicated in the myosteatosis formation.^6^, ^37^, ^38^, ^39^ Notably, these phenomena are associated with enhanced inflammatory milieu,^6^, ^39^ suggesting that the individual inflammatory status may identify distinct myosteatosis pathophysiology that could modulate survival outcomes. In accordance with this hypothesis, we observed that the combination of low systemic inflammation with myosteatosis revealed a subgroup with improved prognosis. These results indicate that intramuscular fat depots that are not associated with cachexia‐mediated inflammation may be a protective factor. Moreover, low visceral fat content worsens prognosis 40 and a recent report showed that diabetes was independently associated with better prognosis in LAEC subjects.^4^ Consistently, our results demonstrate that patients with myosteatosis presented increased adipose tissue in visceral, subcutaneous, and muscular areas. Although no patient presented visceral obesity, analysis of high visceral fat area and VFI as continuous variables showed that they were independently associated with decreased OS. Therefore, these data also suggest that the presence of myosteatosis may be a surrogate marker of adipose tissue depots in LAEC patients.

The thrifty metabolic phenotype hypothesis is currently accepted as the individual ability of increasing or decreasing their energy conservation machinery during famine and overfeeding circumstances.^41^, ^42^, ^43^ In accordance, one of the best methods to predict individual propensity to weight gain is to measure energy expenditure in individuals submitted to low protein overfeeding, therefore individuals who more efficiently decrease energy expenditure during low energy intake tend to gain more weight.^42^, ^44^ Our study shows that individuals with mechanical obstruction of the esophagus that accumulated larger amounts of adipose tissue depots without cancer cachexia‐mediated inflammation lived longer. These results are also in agreement with the hibernation theory, a hypothesis that is used to explain the paradox of obesity whereby as high are energy storages more protected are the individual from long periods of fasting and therefore present favorable cancer‐related outcomes.^45^ In aggregate, it is tempting to speculate that obesity paradox promoted by the hibernation hypothesis is more likely to happen in cancer cachexia where reduced ingestion becomes the preponderant factor concomitant to the absence of the overt of cachexia‐mediated inflammation. These data also suggest that individuals with LAEC and a higher capacity to decrease their energy expenditure will have better prognosis. Given that a previous report observed that adipose tissue distribution between subcutaneous and visceral depots may directly influence OS,^46^ further studies are needed to elucidate how cachexia‐mediated inflammation modulate this association.

A large number of studies evaluated the role of sarcopenia on survival outcomes after esophagectomy. In spite of some studies suggesting that sarcopenia had no impact on OS,^47^, ^48^, ^49^ a recent meta‐analysis^15^ showed that sarcopenia is in fact an unfavorable prognostic factor. In contrast, sarcopenia in metastatic esophageal carcinoma setting was not associated with mortality.^16^, ^50^ On the other hand, the prognostic significance of sarcopenia in patients with LAEC is not well established. We found three studies that evaluate this setting of patients. These studies showed that sarcopenia was not associated with mortality in multivariate analysis.^50^, ^51^, ^52^ In accordance with these results, we also did not detect a role for sarcopenia in predicting prognosis in this analysis. Otherwise, we did not observe that a low muscle index, analyzed as a continuous variable, was associated with poor prognosis in contrast to the previous report of Järvinen et al.^50^

Strengths of our study comprise its large sample of subjects submitted to dCRT in a tertiary hospital. As far as we know, this is the largest study in this setting of patients. Furthermore, it involves a unique kind of cancer population that the cachectic phenotype is mainly determined by mechanical obstruction of digestive tract. On the other hand, we have limited information on tumor and lymph node staging since the patients were not submitted to endoscopic ultrasonography. Furthermore, the study's retrospective and single‐centered nature as well as the complexity inherent to the method of defining patient myosteatosis presence limits its generalizability and clinical utilization.

In conclusion, myosteatosis without systemic inflammation predicted favorable prognosis in patients treated with dCRT for LAEC. Further studies that prospectively explore the role of myosteatosis and inflammatory status as a potential modifiable biomarker may help to strategically build nutritional and pharmacologic interventions that ultimately can improve both our understanding of the involved pathophysiological mechanisms as well as the prognosis of esophageal cancer patients.

## CONFLICT OF INTEREST

The authors declare that they have no conflict of interest.

## AUTHOR CONTRIBUTIONS

CTBG, MCLM, DLM, LPS, and FL collected the data. CTBG, MCLM, DLM, LPS, FL, LTM, MCSM, and JBCC discussed and interpreted the results from the study. CTBG and JBCC conceived and wrote the manuscript. All authors critically reviewed and approved the manuscript for submission.

## Data Availability

Data available on request due to privacy/ethical restrictions.
