# Supplementary figures and images for: Myosteatosis in a systemic inflammation‐dependent manner predicts favorable survival outcomes in locally advanced esophageal cancer
Source: Cancer Med. 2019 Oct 1;8(16):6967–76. doi: 10.1002/cam4.2593 (PMC6853837; doi:10.1002/cam4.2593)

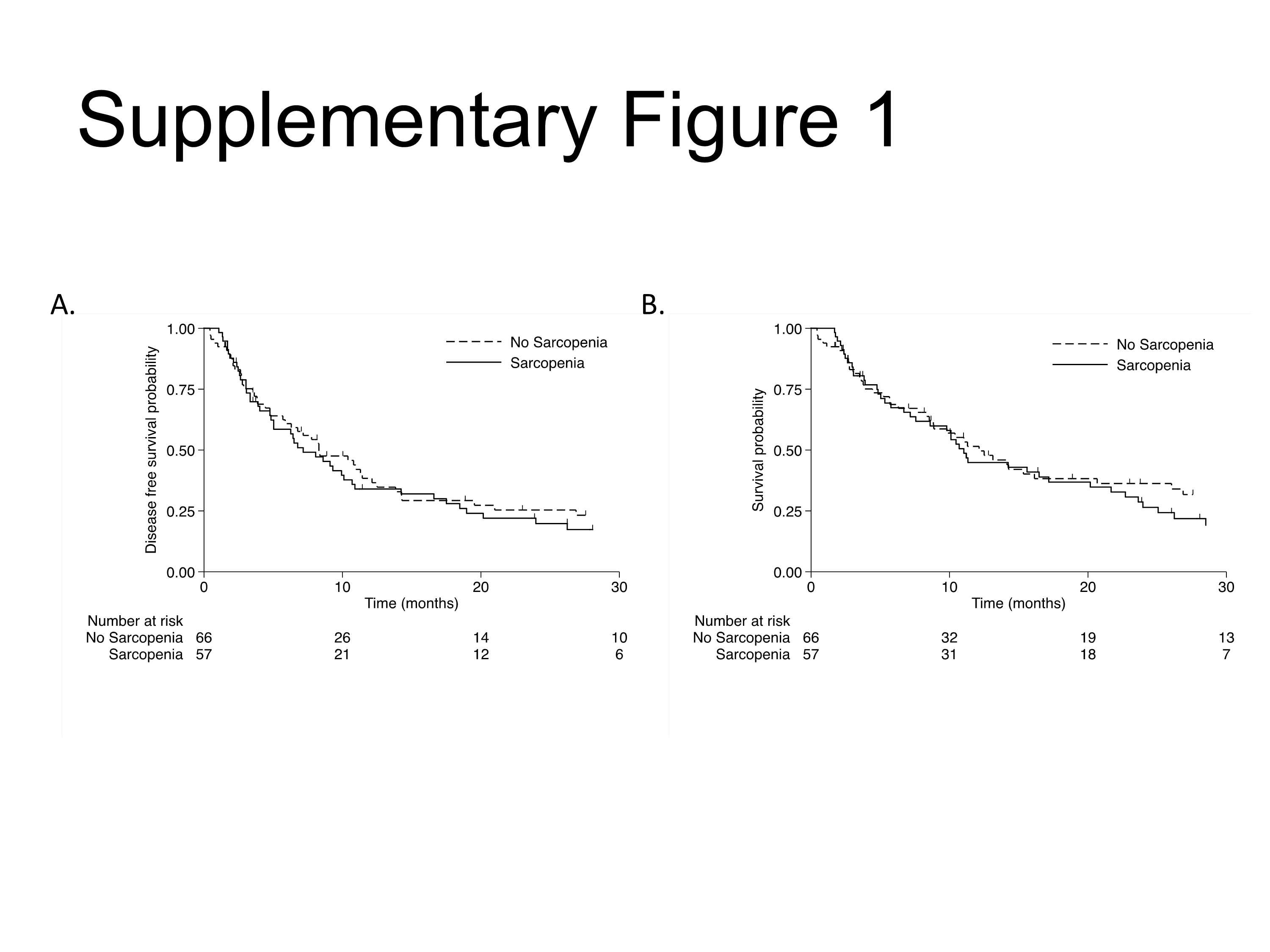

Supplement: Supplementary file 1 [file CAM4-8-6967-s001.TIF]

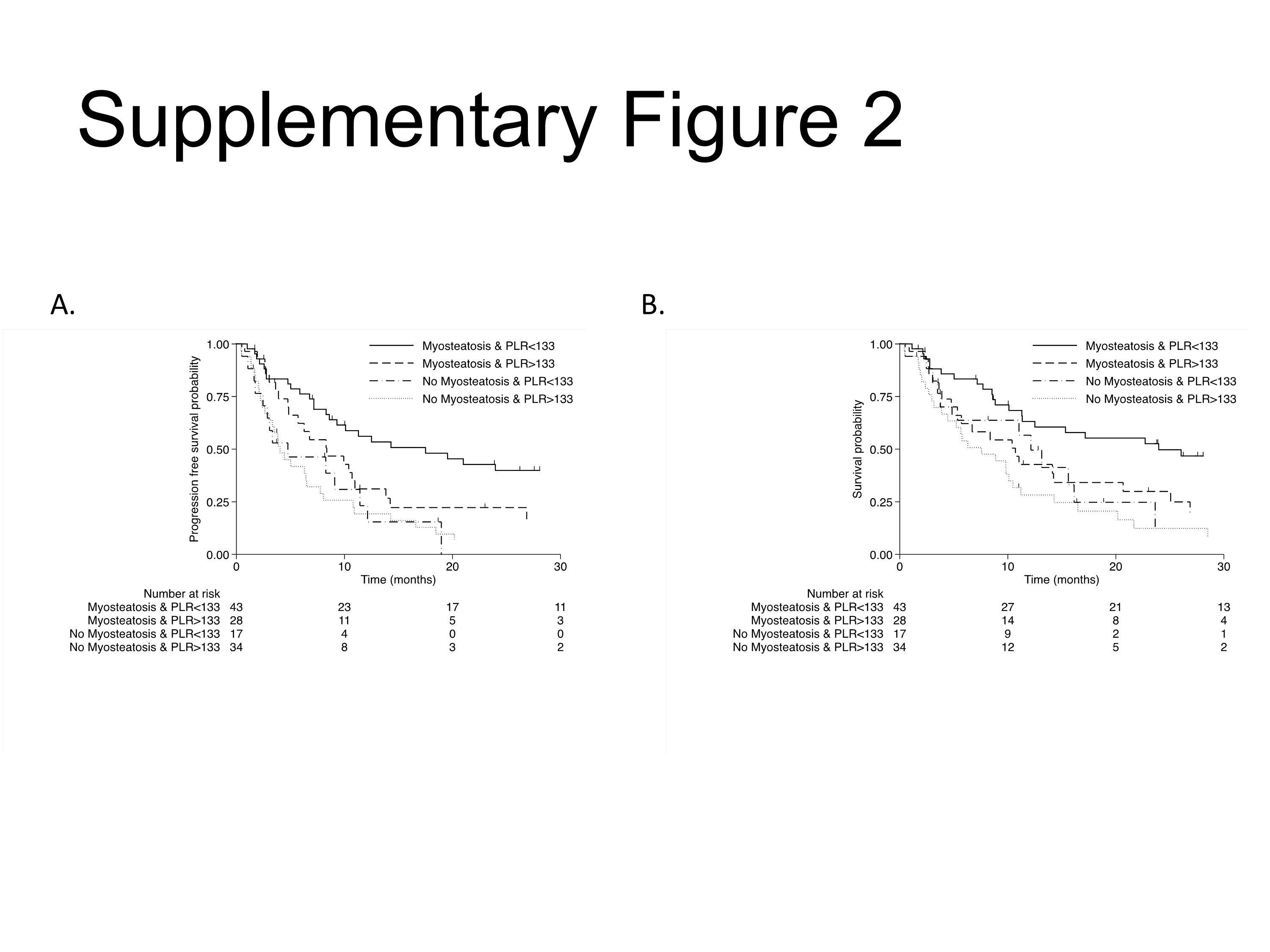

Supplement: Supplementary file 2 [file CAM4-8-6967-s002.TIF]
