# Supplementary material for: Myosteatosis in a systemic inflammation‐dependent manner predicts favorable survival outcomes in locally advanced esophageal cancer
Source: Cancer Med. 2019 Oct 1;8(16):6967–76. doi: 10.1002/cam4.2593 (PMC6853837; doi:10.1002/cam4.2593)
Supplement: Supplementary file 3 [file CAM4-8-6967-s003.docx]

**Supplementary** **Table 1.** Myosteteatosis and survival from date of performed CT.

| **Parameter** | **Non-myosteatosis (n=51)** | **Myosteatosis (n=72)** | ***P v*alue** |
| --- | --- | --- | --- |
| **Progression free survival** |  |  |  |
| # Events / at risk | 44/51 | 53/72 |  |
| Median (months) | 5.2 | 12.3 |  |
| Age-adjusted | Referent | 0.52 (0.34⎼0.79) | 0.002 |
| Adjusted^a^ | Referent | 0.56 (0.36⎼0.88) | 0.011 |
| **Overall survival** |  |  |  |
| # Events / at risk | 41/51 | 49/72 |  |
| Median (months) | 8.9 | 14.2 |  |
| Unadjusted | Referent | 0.59 (0.38⎼0.91) | 0.016 |
| Adjusted^b^ | Referent | 0.59 (0.37-0.93) | 0.024 |

^a^ Cox model adjusted for age (continuous), weight loss (<5%, 5-9.9 or >9), BMI (<18.5, 18.5-24.9, 25-30 or >30) and ECOG (0, 1 or 2).

Abbreviations: BMI: Body Mass Index; ECOG: Eastern Cooperative Oncology Group Performance.
