# Supplementary material for: Myosteatosis in a systemic inflammation‐dependent manner predicts favorable survival outcomes in locally advanced esophageal cancer
Source: Cancer Med. 2019 Oct 1;8(16):6967–76. doi: 10.1002/cam4.2593 (PMC6853837; doi:10.1002/cam4.2593)
Supplement: Supplementary file 4 [file CAM4-8-6967-s004.docx]

**Supplementary** **Table 2.** Univariate and multivariate COX regression of body composition and inflammatory indexes.

|  | | **Progression free survival** | | | | | | | |  | **Overall Survival** | | | | | | |
| --- | --- | --- | --- | --- | --- | --- | --- | --- | --- | --- | --- | --- | --- | --- | --- | --- | --- |
|  | | **Univariate analysis** | | |  | | **Multivariate analysis** | | |  | **Univariate analysis** | | |  | **Multivariate analysis** | | |
|  | **HR** | | **95% CI** | ***P* value** |  | **HR** | | **95% CI** | ***P* value** |  | **HR** | **95% CI** | ***P* value** |  | **HR** | **95% CI** | ***P* value** |
| **Skeletal Muscle** |  | |  |  |  |  | |  |  |  |  |  |  |  |  |  |  |
| Area (cm^2^) | 0.99 | | 0.98⎼1.00 | 0.053 |  | 1.00 | | 0.99⎼1.01 | 0.878 |  | 0.99 | 0.98⎼1.00 | 0.074 |  | 1.00 | 0.99⎼1.01 | 0.652 |
| Mean MA (HU) | 1.05 | | 1.02⎼1.07 | <0.001 |  | 1.03 | | 1.01⎼1.06 | 0.013 |  | 1.04 | 1.02⎼1.06 | 0.001 |  | 1.03 | 1.01⎼1.06 | 0.024 |
| SMI (cm^2^/m^2^) | 0.97 | | 0.95⎼1.00 | 0.061 |  | 1.00 | | 0.98⎼1.00 | 0.804 |  | 0.98 | 0.95⎼1.01 | 0.164 |  | 1.00 | 0.97⎼1.04 | 0.806 |
| Sarcopenia | 1.11 | | 0.71⎼1.66 | 0.606 |  | 0.93 | | 0.59⎼1.46 | 0.752 |  | 1.17 | 0.78⎼1.78 | 0.450 |  | 1.04 | 0.65⎼1.65 | 0.882 |
| **Adipose Tissue** |  | |  |  |  |  | |  |  |  |  |  |  |  |  |  |  |
| Visceral, area (cm^2^) | 0.99 | | 0.991⎼0.997 | <0.001 |  | 0.99 | | 0.990⎼0.997 | 0.003 |  | 0.99 | 0.991⎼0.998 | 0.003 |  | 0.99 | 0.990⎼0.998 | 0.012 |
| VFI (cm^2^/m^2^) | 0.98 | | 0.97⎼0.99 | <0.001 |  | 0.98 | | 0.97⎼0.99 | 0.004 |  | 0.99 | 0.978⎼0.996 | 0.004 |  | 0.99 | 0.976⎼0.998 | 0.018 |
| Subcutaneous, area (cm^2^) | 0.99 | | 0.988⎼0.997 | 0.001 |  | 0.99 | | 0.99⎼1.00 | 0.089 |  | 0.99 | 0.990⎼0.999 | 0.014 |  | 1.00 | 0.99⎼1.00 | 0.239 |
| SFI (cm^2^/m^2^) | 0.98 | | 0.97⎼0.99 | 0.003 |  | 0.99 | | 0.97⎼1.00 | 0.152 |  | 0.99 | 0.975⎼0.998 | 0.025 |  | 0.99 | 0.98⎼1.01 | 0.373 |
| Intramuscular, area (cm^2^) | 0.93 | | 0.90⎼0.97 | <0.001 |  | 0.95 | | 0.91⎼0.99 | 0.019 |  | 0.95 | 0.92⎼0.98 | 0.004 |  | 0.96 | 0.92⎼1.00 | 0.069 |
| **Inflammatory indexes** |  | |  |  |  |  | |  |  |  |  |  |  |  |  |  |  |
| NLR (<2.8) | 0.55 | | 0.37⎼0.83 | 0.004 |  | 0.64 | | 0.42⎼0.98 | 0.042 |  | 0.51 | 0.33⎼0.78 | 0.002 |  | 0.57 | 0.37⎼0.89 | 0.014 |
| PLR (<133) | 0.58 | | 0.38⎼0.87 | 0.008 |  | 0.69 | | 0.44⎼1.06 | 0.089 |  | 0.51 | 0.33⎼0.79 | 0.002 |  | 0.57 | 0.36⎼0.90 | 0.015 |

^a^ Cox model adjusted for age (continuous), weight loss (<5%, 5-9.9 or >9), BMI (<18.5, 18.5-24.9, 25-30 or >30) and ECOG (0, 1 or 2).

Abbreviations: CI: Confidence Interval; HR: Hazard Ratio; HU: Hounsfield Units; IQR: Interquartile Range; MA: Muscle Attenuation; NLR: Neutrophil to Lymphocyte Ratio; SD: Standard Deviation; SFI: Subcutaneous Fat Index; SMI: Skeletal Muscle Index; VFI: Visceral Fat Index.
