# Supplementary material for: Myosteatosis in a systemic inflammation‐dependent manner predicts favorable survival outcomes in locally advanced esophageal cancer
Source: Cancer Med. 2019 Oct 1;8(16):6967–76. doi: 10.1002/cam4.2593 (PMC6853837; doi:10.1002/cam4.2593)
Supplement: Supplementary file 5 [file CAM4-8-6967-s005.docx]

**Supplementary Table 3.** Selected characteristics according to NLR of esophageal cancer patients with myosteatosis

| **Characteristic** | **NLR<2.8 (n=44)** | **NLR>2.8 (n=28)** | ***P v*alue** |
| --- | --- | --- | --- |
| **Age, mean (SD), y** | 61.2 (12.4) | 62.1 (12.4) | 0.77 |
| **Sex, № (%)** |  |  |  |
| Male | 40 (90.9) | 23 (82.1) | 0.27 |
| Female | 4 (9.0) | 5 (17.9) |  |
| **Body mass index (kg/m^2^), № (%)** |  |  |  |
| <18.5 | 8 (18.2) | 8 (28.6) | 0.57 |
| 18.5-24.9 | 28 (63.6) | 17 (61.7) |  |
| 25-30 | 7 (15.9) | 2 (7.1) |  |
| >30 | 1 (2.3) | 1 (3.6) |  |
| **Weight loss, № (%)** |  |  |  |
| <5 | 4 (9.0) | 1 (3.6) | 0.30 |
| 5-9.9 | 13 (29.6) | 5 (17.8) |  |
| >10 | 27 (61.4) | 22 (78.6) |  |
| **Hypertension, № (%)** | 16 (36.4) | 9 (32.1) | 0.71 |
| **Dyslipidemia , № (%)** | 1 (2.3) | 1 (3.6) | 0.74 |
| **Diabetes , № (%)** | 2 (4.6) | 1 (3.6) | 0.84 |
| **Histology, № (%)** |  |  |  |
| Adenocarcinoma | 5 (11.4) | 3 (10.7) | 0.93 |
| Squamous cell carcinoma | 39 (88.6) | 25 (89.3) |  |
| **Tumor location, № (%)** |  |  |  |
| Upper third | 6 (13.6) | 6 (21.4) | 0.13 |
| Middle third | 28 (63.6) | 11 (39.3) |  |
| Lower third | 10 (22.7) | 11 (39.3) |  |
| **Chemotherapy, № (%)** |  |  |  |
| 5-Fluorouracil + Cisplatin | 10 (22.7) | 2 (7.1) | 0.11 |
| Carboplatin + Paclitaxel | 34 (77.3) | 25 (89.3) |  |
| Others | 0 (0.0) | 1 (3.6) |  |
| **Toxicity grade III-IV, № (%)** |  |  |  |
| No | 32 (72.7) | 18 (64.3) | 0.45 |
| Yes | 12 (27.3) | 10 (35.7) |  |
| **ECOG, № (%)** |  |  |  |
| 0_ | 28 (63.6) | 8 (29.6) | 0.01 |
| 1_ | 15 (34.1) | 19 (70.4) |  |
| 2_ | 1 (2.3) | 0 (0.0) |  |

Abbreviations: ECOG: Eastern Cooperative Oncology Group Performance; SD: Standard Deviation

**Supplementary Table 4.** Selected characteristics according to PLR of esophageal cancer patients with myosteatosis

| **Characteristic** | **PLR<133 (n=43)** | **PLR>133 (n=28)** | ***P v*alue** |
| --- | --- | --- | --- |
| **Age, mean (SD), y** | 61.7 (13.8) | 61.4 (10.0)  (10.2) | 0.94 |
| **Sex, № (%)** |  |  |  |
| Male | 38 (88.4) | 24 (85.7) | 0.74 |
| Female | 5 (11.6) | 4 (14.3) |  |
| **Body mass index (kg/m^2^), № (%)** |  |  |  |
| <18.5 | 8 (18.6) | 8 (28.6) | 0.76 |
| 18.5-24.9 | 28 (65.1) | 16 (57.1) |  |
| 25-30 | 6 (14.0) | 3 (10.7) |  |
| >30 | 1 (2.3) | 1 (3.6) |  |
| **Weight loss, № (%)** |  |  |  |
| <5 | 4 (9.3) | 1 (3.6) | 0.49 |
| 5-9.9 | 14 (32.6) | 3 (10.7) |  |
| >10 | 25 (58.1) | 24 (85.7) |  |
| **Hypertension, № (%)** | 15 (34.9) | 9 (32.1) | 0.81 |
| **Dyslipidemia , № (%)** | 2 (4.7) | 0 (0.0) | 0.25 |
| **Diabetes , № (%)** | 2 (4.7) | 1 (3.6) | 0.83 |
| **Histology, № (%)** |  |  |  |
| Adenocarcinoma | 6 (14.0) | 1 (3.6) | 0.15 |
| Squamous cell carcinoma | 37 (86.0) | 27 (96.4) |  |
| **Tumor location, № (%)** |  |  |  |
| Upper third | 6 (13.9) | 6 (21.4) | 0.69 |
| Middle third | 24 (55.8) | 15 (53.6) |  |
| Lower third | 13 (30.2) | 7 (25.0) |  |
| **Chemotherapy, № (%)** |  |  |  |
| 5-Fluorouracil + Cisplatin | 9 (20.9) | 3 (10.7) | 0.26 |
| Carboplatin + Paclitaxel | 34 (79.1) | 24 (85.7) |  |
| Others | 0 (0.0) | 1 (3.6) |  |
| **Toxicity grade III-IV, № (%)** |  |  |  |
| No | 29 (67.4) | 21 (75.0) | 0.49 |
| Yes | 14 (32.6) | 7 (25.0) |  |
| **ECOG, № (%)** |  |  |  |
| 0_ | 27 (62.8) | 9 (33.3) | 0.04 |
| 1_ | 16 (37.2) | 17 (63.0) |  |
| 2_ | 0 (0.0) | 1 (3.7) |  |

Abbreviations: ECOG: Eastern Cooperative Oncology Group Performance; SD: Standard Deviation
