# Supplementary material for: Myosteatosis in a systemic inflammation‐dependent manner predicts favorable survival outcomes in locally advanced esophageal cancer
Source: Cancer Med. 2019 Oct 1;8(16):6967–76. doi: 10.1002/cam4.2593 (PMC6853837; doi:10.1002/cam4.2593)
Supplement: Supplementary file 6 [file CAM4-8-6967-s006.docx]

**Supplementary Table 5.** Myosteatosis, platelet to lymphocyte ratio and survival from date of treatment start.

|  | **Progression free survival** | | | | |  | **Overall Survival** | | | | |
| --- | --- | --- | --- | --- | --- | --- | --- | --- | --- | --- | --- |
|  | **No Myosteatosis** | |  | **Myosteatosis** | |  | **No Myosteatosis** | |  | **Myosteatosis** | |
|  | **PLR<132** | **PLR>132** |  | **PLR<132** | **PLR>132** |  | **PLR<132** | **PLR>132** |  | **PLR<132** | **PLR>132** |
| # Events / at risk | 15/18 | 30/34 |  | 29/43 | 22/27 |  | 14/18 | 28/34 |  | 25/43 | 22/27 |
| Median (months) | 5.7 | 4.0 |  | 17.5 | 8.3 |  | 13.1 | 7.6 |  | 24.0 | 10.4 |
| HR | 1.214 | |  | 0.550 | |  | 0.942 | |  | 0.450 | |
| 95% CI | 0.607⎼2.427 | |  | 0.292⎼1.034 | |  | 0.464⎼1.912 | |  | 0.236⎼0.857 | |
| *P* | 0.583 | |  | 0.064 | |  | 0.868 | |  | 0.015 | |

Cox model adjusted for age (continuous), weight loss (<5%, 5-9.9 or >9), BMI (<18.5, 18.5-24.9, 25-30 or >30) and ECOG (0, 1 or 2).  Abbreviations: BMI: Body Mass Index; CI: ConfidenceInterval; ECOG: Eastern Cooperative Oncology Group Performance; PLR: platelet to lymphocyte ratio.
