# Supplementary material for: Myosteatosis in a systemic inflammation‐dependent manner predicts favorable survival outcomes in locally advanced esophageal cancer
Source: Cancer Med. 2019 Oct 1;8(16):6967–76. doi: 10.1002/cam4.2593 (PMC6853837; doi:10.1002/cam4.2593)
Supplement: Supplementary file 7 [file CAM4-8-6967-s007.docx]

**Figure S1.** Progression free survival (A) and overall survival (B) in patients with and without sarcopenia with locally advanced esophageal cancer treated with definitive chemoradiotherapy

**Figure S2.** Progression free survival (A) and overall survival (B) according to platelet-to-lymphocyte ratio and myosteatosis in patients with locally advanced esophageal cancer treated with definitive chemoradiotherapy
